# Effects of the COVID-19 pandemic on pediatric trauma in Southern California

**DOI:** 10.1007/s00383-021-05050-6

**Published:** 2021-12-01

**Authors:** Eric O. Yeates, Areg Grigorian, Morgan Schellenberg, Natthida Owattanapanich, Galinos Barmparas, Daniel Margulies, Catherine Juillard, Kent Garber, Henry Cryer, Areti Tillou, Sigrid Burruss, Liz Penaloza-Villalobos, Ann Lin, Ryan Arthur Figueras, Raul Coimbra, Megan Brenner, Todd Costantini, Jarrett Santorelli, Terry Curry, Diane Wintz, Walter L. Biffl, Kathryn B. Schaffer, Thomas K. Duncan, Casey Barbaro, Graal Diaz, Arianne Johnson, Justine Chinn, Ariana Naaseh, Amanda Leung, Christina Grabar, Jeffry Nahmias

**Affiliations:** 1 Department of Surgery, University of California, Irvine (UCI), 333 The City Blvd West, Suite 1600, Orange, CA 92868-3298 USA; 2 Department of Surgery, University of Southern California (USC), Los Angeles, CA USA; 3 Department of Surgery, Cedars-Sinai Medical Center, Los Angeles, CA USA; 4 Department of Surgery, University of California, Los Angeles (UCLA), Los Angeles, CA USA; 5 Department of Surgery, Loma Linda University, Loma Linda, CA USA; 6 Riverside University Health System Medical Center, Moreno Valley, CA USA; 7 Department of Surgery, University of California, Riverside/Riverside University Health System, Moreno Valley, CA USA; 8 Department of Surgery, University of California, San Diego (UCSD), San Diego, CA USA; 9 Department of Surgery, Sharp Memorial Hospital, San Diego, CA USA; 10 Trauma Department, Scripps Memorial Hospital La Jolla, La Jolla, CA USA; 11 Department of Surgery, Ventura County Medical Center, Ventura, CA USA; 12 Santa Barbara Cottage Hospital, Cottage Health Research Institute, Santa Barbara, CA USA

**Keywords:** Pediatric, Trauma, COVID-19, Pandemic, Penetrating

## Abstract

**Purpose:**

The COVID-19 pandemic resulted in increased penetrating trauma and decreased length of stay (LOS) amongst the adult trauma population, findings important for resource allocation. Studies regarding the pediatric trauma population are sparse and mostly single-center. This multicenter study examined pediatric trauma patients, hypothesizing increased penetrating trauma and decreased LOS after the 3/19/2020 stay-at-home (SAH) orders.

**Methods:**

A multicenter retrospective analysis of trauma patients ≤ 17 years old presenting to 11 centers in California was performed. Demographic data, injury characteristics, and outcomes were collected. Patients were divided into three groups based on injury date: 3/19/2019–6/30/2019 (CONTROL), 1/1/2020–3/18/2020 (PRE), 3/19/2020–6/30/2020 (POST). POST was compared to PRE and CONTROL in separate analyses.

**Results:**

1677 patients were identified across all time periods (CONTROL: 631, PRE: 479, POST: 567). POST penetrating trauma rates were not significantly different compared to both PRE (11.3 vs. 9.0%, *p* = 0.219) and CONTROL (11.3 vs. 8.2%, *p* = 0.075), respectively. POST had a shorter mean LOS compared to PRE (2.4 vs. 3.3 days, *p* = 0.002) and CONTROL (2.4 vs. 3.4 days, *p* = 0.002). POST was also not significantly different than either group regarding intensive care unit (ICU) LOS, ventilator days, and mortality (all *p* > 0.05).

**Conclusions:**

This multicenter retrospective study demonstrated no difference in penetrating trauma rates among pediatric patients after SAH orders but did identify a shorter LOS.

## Introduction

The COVID-19 pandemic resulted in stay-at-home (SAH) orders and other mandates designed to slow the spread of disease [1–3]. These restrictions also triggered business closures, economic losses, and psychological stressors that have significantly impacted society [4–11]. These changes have been particularly detrimental to children who have also been impacted by school closures and social isolation [12–16]. Therefore, injury patterns and outcomes within the pediatric trauma population had the potential to change during the pandemic.

Significant shifts within the adult trauma population during COVID-19 are now well described and include an increase in penetrating trauma and drug use, variations in trauma volume based on insurance status, and a shorter length of stay (LOS) [17–26]. However, studies dedicated to the pediatric trauma population are sparse and have been mostly single-center in design [27–34]. One of these studies demonstrated a similar increase in pediatric penetrating trauma, as has been seen with adult trauma, during the COVID-19 pandemic [27]. Another study reporting that child-involved shooting incidents increased in 2020 during the COVID-19 pandemic also supports the possibility that penetrating trauma rates are increasing in the pediatric population [35].

Therefore, we sought to explore the changes in mechanisms and outcomes among the pediatric trauma population during the COVID-19 pandemic in a multicenter study, as these could affect resource allocation in the future. We hypothesized an increase in penetrating trauma rates and a shorter LOS in the Southern California pediatric trauma population after the March 19, 2020 SAH orders [36].

## Methods

A post hoc analysis of pediatric trauma patients presenting to 11 American College of Surgeons (ACS) Level I and II trauma centers in Southern California between 1/1/2020–6/30/2020 and 3/19/2019–6/30/2019 was performed. These 11 centers span 7 counties in Southern California and comprise a mixture of academic and private hospitals that serve mostly urban areas. The study was approved by the Institutional Review Board (IRB) of University of California, Irvine and all participating centers and was deemed exempt from need for consent.

All patients ≤ 17 years old who were either a trauma activation or trauma consult were included in this study. The primary outcome was the rate of penetrating trauma, categorized as either gunshot wounds, stab wounds, or other. The secondary outcome was LOS. Other outcomes were intensive care unit (ICU) admission, ICU LOS, ventilator days, operations performed (tracheostomy, laparotomy, craniectomy/craniotomy, and vascular/endovascular surgery), and mortality. Demographic data were collected which included sex (self-reported), age, race, insurance status (i.e., Medicaid, private, and uninsured), and body mass index (BMI). Other mechanisms of injury recorded included motor vehicle collisions (MVC), pedestrian struck, motorcycle collision (MCC), sports injuries, ground level falls (GLF), falls from height, and assaults. Injury severity score (ISS), serum alcohol positivity, and urine toxicology were also recorded. Vital signs and exam findings on arrival were also collected and included heart rate (HR), respiratory rate (RR), systolic blood pressure (SBP), and Glasgow Coma Scale (GCS) score. Discharge disposition was collected and included home, long-term acute care hospital (LTAC), and acute rehabilitation.

Patients were then divided into three groups based on the date of injury: a historical control from 3/19/2019–6/30/2019 (CONTROL), immediately before the SAH order from 1/1/2020–3/18/2020 (PRE), and after the SAH order 3/19/2020–6/30/2020 (POST). Descriptive statistics were performed for all variables within each group, with categorical variables reported as percentages of their respective group and continuous variables as means with standard deviations. The POST group was compared to the PRE group and the CONTROL group in two separate analyses. This was done to account for both seasonal and annual variations that exist within the trauma population. Additionally, different subgroups including patients 12 years and older, younger than 12 years old, ISS greater than or equal to 10, and ISS less than 10 were created. LOS was compared between the three time periods for each subgroup. Chi-square tests were used to compare categorical variables and Mann–Whitney *U* tests for continuous variables. Next, multivariable logistic regression utilizing time period, age, race, insurance status, and ISS was performed to identify independent risk factors for a LOS longer than 2 days. The adjusted risk of LOS longer than 2 days was reported as an odds ratio (OR) with a 95% confidence interval (CI). *p* values < 0.05 were considered statistically significant. All analyses were performed using IBM SPSS Statistics for Windows (Version 24, IBM Corp., Armonk, NY).

## Results

A total of 1677 trauma patients were identified across the 3 time periods: 631 patients in the CONTROL group, 479 in the PRE group, and 567 in the POST group.

### PRE vs. POST demographics

Compared to the PRE group, the POST group had a higher percentage of white (42.0 vs. 35.9%, *p* = 0.045) and Black (9.3 vs. 5.8%, *p* = 0.035) patients, but a lower percentage of Latino patients (41.1 vs. 50.3%, *p* = 0.003). The POST group also had significantly higher rate of Medicaid (46.9 vs. 31.5%, *p* < 0.001), but lower rate of private insurance (43.0 vs. 56.6%, *p* < 0.001). Otherwise, the two groups were similar regarding sex, age, race, insurance status, and BMI (all p > 0.05) (Table 1).

**Table 1:** Demographics of pediatric trauma patients compared by time period

Characteristic	POST (*n* = 567)	PRE (*n* = 479)	PRE vs POST	CONTROL (*n* = 631)	CONTROL vs POST
			*p* value		*p* value
Male, *n* (%)	366 (64.6%)	293 (61.2%)	0.259	419 (66.4%)	0.501
Age, years, mean ± SD	9.9 ± 5.6	10.0 ± 5.6	0.833	10.1 ± 5.6	0.685
Race, *n* (%)					
White	238 (42.0%)	172 (35.9%)	**0.045**	250 (39.6%)	0.407
Latino	233 (41.1%)	241 (50.3%)	**0.003**	254 (40.3%)	0.768
Black	53 (9.3%)	28 (5.8%)	**0.035**	54 (8.6%)	0.632
Asian	15 (2.6%)	15 (3.1%)	0.639	16 (2.5%)	0.905
Insurance status, *n* (%)					
Medicaid	266 (46.9%)	151 (31.5%)	**< 0.001**^**+**^	216 (34.2%)	**< 0.001**^**+**^
Private	244 (43.0%)	271 (56.6%)	**< 0.001**^**+**^	362 (57.4%)	**< 0.001**^**+**^
Uninsured	22 (3.9%)	30 (6.3%)	0.077	41 (6.5%)	**0.043**
BMI, kg/m^2^, mean ± SD	21.3 ± 6.3	20.8 ± 5.9	0.220	20.1 ± 5.5	**0.006**

Bolded values are significantly different

*SD* standard deviation, *BMI* body mass index, *CONTROL* 3/19/19–6/30/19, *PRE* 1/1/20–3/18/20, *POST* 3/19/20–6/30/20

^**+**^Significantly different in both comparisons

### CONTROL vs. POST demographics

Compared to the CONTROL group, the POST group had a higher rate of Medicaid (46.9 vs. 34.2%, *p* < 0.001), but lower rates of private insurance (43.0 vs. 57.4%, *p* < 0.001) and no insurance (3.9 vs. 6.5%, *p* = 0.043). The POST group also had a higher mean BMI (21.3 vs. 20.1 kg/m^2^, *p* = 0.006). Otherwise, the two groups were similar regarding sex, age, and race (all *p* > 0.05) (Table 1).

### PRE vs. POST injury characteristics, toxicology, and vital signs

Compared to the PRE group, the POST group had a similar rate of penetrating trauma (11.3 vs. 9.0%, *p* = 0.219) including both gunshot and stab wounds individually (both *p* > 0.05), but a lower rate of MVC (21.2 vs. 27.3%, *p* = 0.020). Otherwise, the groups were similar in terms of mechanism of injury, alcohol/drug positivity, ISS, and vital signs on arrival (all *p* > 0.05) (Table 2).

**Table 2:** Injury characteristics, toxicology, and vital signs of pediatric trauma patients compared by time period

Characteristic	POST (*n* = 567)	PRE (*n* = 479)	PRE vs POST	CONTROL (*n* = 631)	CONTROL vs POST
			*p* value		*p* value
Mechanism of injury, *n* (%)					
Blunt	503 (88.7%)	436 (91.0%)	0.219	579 (91.8%)	0.075
Ground level fall	87 (15.3%)	64 (13.4%)	0.363	91 (14.4%)	0.654
Fall from height	76 (13.4%)	49 (10.2%)	0.115	82 (13.0%)	0.835
Pedestrian struck	38 (6.7%)	43 (9.0%)	0.170	65 (10.3%)	**0.027**
Motorcycle collision	22 (3.9%)	17 (3.5%)	0.778	48 (7.6%)	**0.006**
Motor vehicle collision	120 (21.2%)	131 (27.3%)	**0.020**	140 (22.2%)	0.668
Assault	30 (5.3%)	21 (4.4%)	0.497	48 (7.6%)	0.105
Sports injury	62 (10.9%)	44 (9.2%)	0.350	61 (9.7%)	0.470
Penetrating	64 (11.3%)	43 (9.0%)	0.219	52 (8.2%)	0.075
Gunshot	25 (4.4%)	22 (4.6%)	0.886	27 (4.3%)	0.912
Stab wound	13 (2.3%)	8 (1.7%)	0.474	13 (2.1%)	0.783
Alcohol positive, *n* (%)	53 (9.3%)	44 (9.2%)	0.928	58 (9.2%)	0.926
Urine toxicology positive, *n* (%)	82 (14.4%)	77 (16.1%)	0.469	72 (11.4%)	0.115
ISS, mean ± SD	7.3 ± 7.5	7.4 ± 8.0	0.920	7.8 ± 9.5	0.594
Vitals on admission, mean ± SD					
Systolic blood pressure, mmHg	122.2 ± 18.1	121.5 ± 18.2	0.556	120.4 ± 17.9	**0.045**
Respiratory rate, breaths per minute	21.1 ± 5.8	21.7 ± 6.5	0.208	21.1 ± 7.4	0.354
Heart rate, beats per minute	108.1 ± 25.4	105.8 ± 25.5	0.100	105.1 ± 27.1	**0.010**
GCS score	14.1 ± 2.7	14.1 ± 2.7	0.327	14.1 ± 2.8	0.704

Bolded values are significantly different

*ISS* injury severity score, *SD* standard deviation, *GCS* Glasgow coma scale, *CONTROL* 3/19/19–6/30/19, *PRE* 1/1/20–3/18/20, *POST* 3/19/20–6/30/20

### CONTROL vs. POST injury characteristics, toxicology, and vital signs

Compared to the CONTROL group, the POST group had a similar penetrating trauma rate (11.3 vs. 8.2%, *p* = 0.075) including both gunshot and stab wounds individually (both *p* > 0.05). However, the POST group had lower rates of pedestrians struck (6.7 vs. 10.3%, *p* = 0.027) and MCC (3.9 vs. 7.6%, *p* = 0.006). The POST group had a higher mean SBP (122.2 vs. 120.4 mmHg, *p* = 0.045) and HR (108.1 vs. 105.1 beats per minute, *p* = 0.010) on arrival. Otherwise, the two groups were similar with regards to mechanism of injury, alcohol/drug positivity, and vital signs on arrival (all *p* > 0.05) (Table 2).

### PRE vs. POST Outcomes

Compared to the PRE group, the POST group had a shorter mean LOS (2.4 vs. 3.3 days, *p* = 0.002). The POST group also had higher rates of discharge to home (80.6 vs. 75.6%, *p* = 0.049), but lower rates of discharge to acute rehabilitation (1.8 vs. 3.8%, *p* = 0.047). Otherwise, the two groups were similar in terms of ICU admission, ICU LOS, ventilator days, operations, discharge disposition, and mortality (all *p* > 0.05) (Table 3).

**Table 3:** Outcomes of pediatric trauma patients compared by time period

Outcome	POST (*n* = 567)	PRE (*n* = 479)	PRE vs POST	CONTROL (*n* = 631)	CONTROL vs POST
			*p* value		*p* value
LOS, days, mean ± SD	2.4 ± 2.9	3.3 ± 5.8	**0.002**^**+**^	3.4 ± 6.2	**0.002**^**+**^
ICU admission, *n* (%)	120 (21.2%)	122 (25.5%)	0.010	148 (23.5%)	0.342
ICU LOS, days, mean ± SD	0.6 ± 1.7	1.1 ± 4.2	0.103	1.0 ± 3.3	0.226
Mechanical ventilation, *n* (%)	42 (7.4%)	41 (8.6%)	0.492	47 (7.4%)	0.978
Ventilator, days, mean ± SD	0.3 ± 1.2	0.6 ± 3.1	0.451	0.5 ± 2.4	0.872
Operations, *n* (%)					
Tracheostomy	3 (0.5%)	4 (0.8%)	0.545	4 (0.6%)	0.812
Laparotomy	6 (1.1%)	11 (2.3%)	0.115	21 (3.3%)	**0.008**
Craniectomy/craniotomy	10 (1.8%)	14 (2.9%)	0.212	16 (2.5%)	0.360
Vascular/endovascular	2 (0.4%)	3 (0.6%)	0.523	2 (0.3%)	0.915
Discharge disposition, *n* (%)					
Home	457 (80.6%)	362 (75.6%)	**0.049**	507 (80.3%)	0.903
Long-term acute care hospital	5 (0.9%)	4 (0.8%)	0.935	6 (1.0%)	0.900
Acute rehabilitation	10 (1.8%)	18 (3.8%)	**0.047**	18 (2.9%)	0.213
Mortality, *n* (%)	11 (1.9%)	11 (2.3%)	0.689	19 (3.0%)	0.236

Bolded values are significantly different

*LOS* length of stay, *ICU* intensive care unit, SD standard deviation, *CONTROL* 3/19/19–6/30/19, *PRE* 1/1/20–3/18/20, *POST* 3/19/20–6/30/20

^**+**^Significantly different in both comparisons

### CONTROL vs. POST Outcomes

Compared to the CONTROL group, the POST group had a shorter mean LOS (2.4 vs. 3.4 days, *p* = 0.002) and a lower rate of laparotomy (2.0 vs. 2.8%, *p* = 0.002). Otherwise, the two groups were similar in terms of ICU admission, ICU LOS, ventilator days, discharge disposition, and mortality (all *p* > 0.05) (Table 3).

### Mechanisms of injury of pediatric trauma patients by time period

**Fig. 1:**
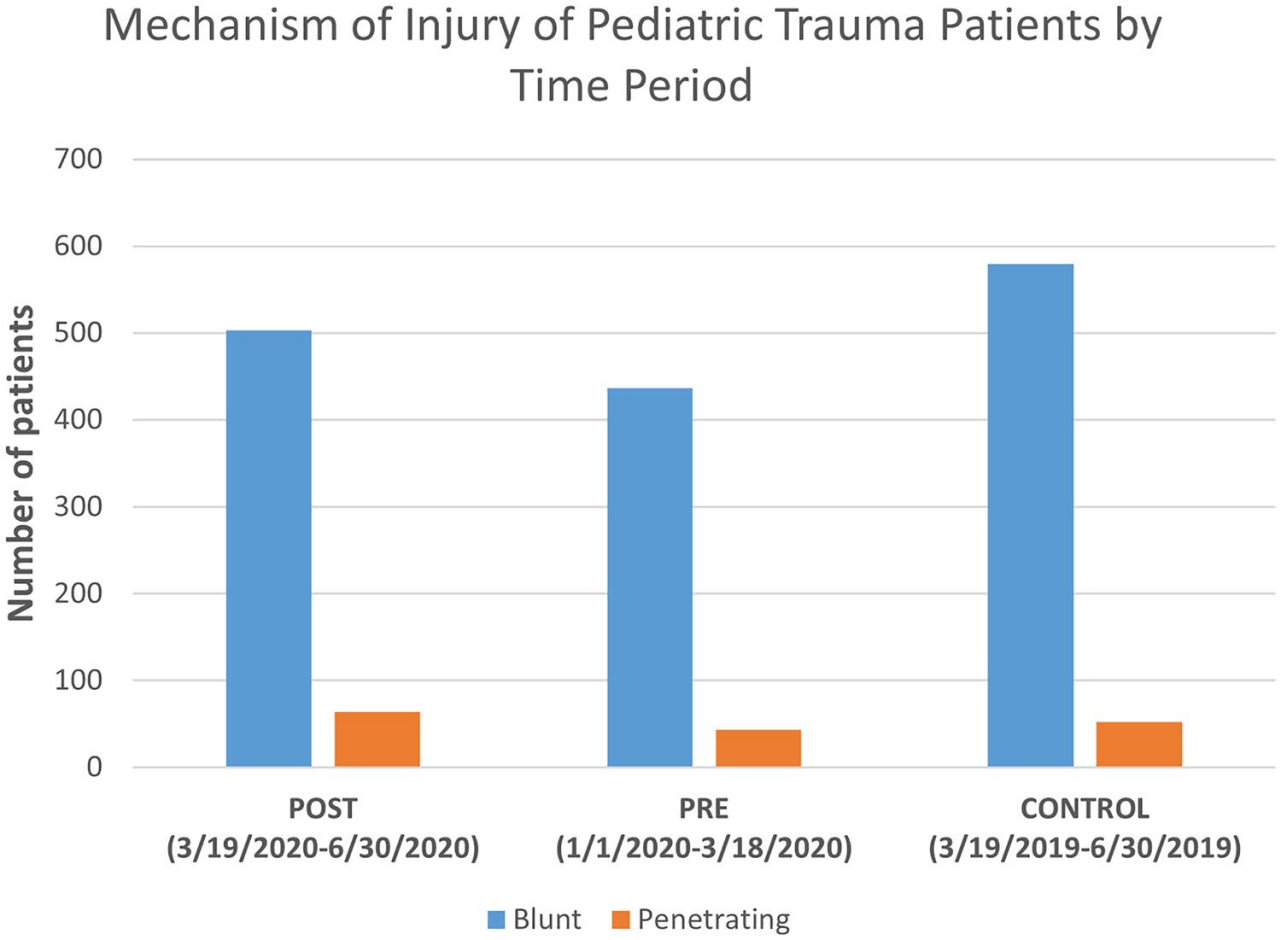
Mechanism of injury of pediatric trauma patients by time period. Bar graph with time periods, separated by blunt and penetrating mechanism of injury, displayed on the *x*-axis and the number of patients displayed on the *y*-axis

In the POST group, there were 503 patients who sustained blunt and 64 who sustained penetrating injuries. In the PRE group, 436 sustained blunt and 43 sustained penetrating injuries. In the CONTROL group, 579 patients sustained blunt injuries and 52 sustained penetrating injuries (Fig. 1).

### Length of stay for different pediatric trauma subgroups compared by time period

Among patients 12 years and older, mean LOS was significantly shorter in the POST group compared to both the PRE and CONTROL groups, respectively (2.5 vs 3.5 days, *p* = 0.026) (2.5 vs 3.5 days, *p* = 0.032). In patients younger than 12 years, LOS was similarly shorter in the POST group (2.2 vs 3.1 days, *p* = 0.022) (2.2 vs 3.2 days, *p* = 0.003). Among patients with an ISS greater than or equal to 10, mean LOS was significantly shorter in the POST group compared to both the PRE and CONTROL groups, respectively (4.5 vs 7.8 days, *p* = 0.004) (4.5 vs 8.9 days, *p* < 0.001). In patients with an ISS less than 10, LOS of the POST group was not significantly different than that of the other two time periods (*p* > 0.05) (Table 4).

**Table 4:** Length of stay for different pediatric trauma subgroups compared by time period

Subgroup	POST	PRE	PRE vs POST		CONTROL vs POST
			*p* value	CONTROL	*p* value
Age ≥ 12 years, days, mean ± SD	2.5 ± 3.2	3.5 ± 5.9	**0.026**	3.5 ± 7.3	**0.032**
Age < 12 years, days, mean ± SD	2.2 ± 2.6	3.1 ± 5.8	**0.022**	3.2 ± 4.9	**0.003**
ISS ≥ 10, days, mean ± SD	4.5 ± 4.7	7.8 ± 10.3	**0.004**	8.9 ± 12.6	**< 0.001**
ISS < 10, days, mean ± SD	1.8 ± 2.0	2.0 ± 2.1	0.141	2.1 ± 2.1	0.089

Bolded values are significantly different

*ISS* injury severity score, SD standard deviation, *CONTROL* 3/19/19–6/30/19, *PRE* 1/1/20–3/18/20, *POST* 3/19/20–6/30/20

^**+**^Significantly different in both comparisons

### Multivariable logistic regression analysis for risk of LOS longer than 2 days in pediatric trauma patients

On multivariable logistic regression analysis, an ISS greater than 15 was an independent associated risk factor for LOS longer than 2 days (OR = 4.99, CI 3.42–7.29, *p* < 0.001). Presenting during the POST period was associated with a decreased risk of having a LOS longer than 2 days (OR = 0.66, CI 0.54–0.82, *p* = < 0.001) (Table 5).

**Table 5:** Multivariable logistic regression analysis for risk of length of stay (LOS) longer than 2 days in pediatric trauma patients

Risk factor	OR	CI	*p* value
POST	0.66	0.54–0.82	< 0.001
Age ≥ 12 years	0.87	0.71–1.10	0.165
Not white	1.04	0.85–1.28	0.707
Not private insurance	1.13	0.92–1.38	0.246
ISS > 15	4.99	3.42–7.29	< 0.001

*OR* odds ratio, *CI* confidence interval, *POST* 3/19/20–6/30/20, *ISS* injury severity score

## Discussion

There are few studies detailing the effects the COVID-19 pandemic has had on pediatric trauma mechanisms and outcomes. This retrospective multicenter study across Southern California found no difference in penetrating trauma rates after SAH orders including analyses on gunshot and stab wounds specifically. However, there was a shorter mean LOS in the POST cohort compared to both the PRE and CONTROL groups. There were no differences between cohorts in terms of other parameters such as drug and alcohol positivity and insurance status.

A rise in community violence during COVID-19 has resulted in a parallel increase in penetrating trauma rates among adults, but has not definitively affected the pediatric trauma population [37–40]. This current multicenter study found a slightly higher although statistically similar penetrating trauma rate among pediatric patients before and after SAH orders. Though some of our other findings, including a decrease in MVC, MCC, and pedestrians struck, are similar to that of prior studies, our findings regarding penetrating trauma oppose those reported by Sherman et al. who found an increase in penetrating trauma among pediatric patients during COVID-19 [27]. However, Sherman et al.’s study had a number of limitations including the inclusion of 18- and 19-year-old patients and the classification of animal bites (which comprised 42% of penetrating trauma in the study) as penetrating trauma which has different trauma and societal implications. Moreover, only a single comparison was made to the mean of the prior 4 years, which does not take into account there may have been a pre-existing annual trend for increased penetrating trauma prior to the pandemic. In contrast, our study compares the COVID-19 data to the year prior, as well as the time frame immediately preceding the pandemic to account for this potential confounder. Furthermore, findings of this multicenter study suggest that the pediatric trauma population was affected differently than the adult population by COVID-19 and SAH orders. This could be due to different pandemic-related stressors and children being less affected by the violence that has risen in the community. These findings could be useful to pediatric trauma centers when planning for future pandemics, as they could improve resource allocation.

Predicting LOS in pediatric trauma patients is important for resource management, especially during a pandemic [41]. This study identified a 1 day shorter mean LOS during the COVID-19 pandemic, compared to both the time period immediately prior and a historical control. Furthermore, we were also able to show that presenting during the COVID-19 pandemic was associated with a decreased risk of having a hospital stay longer than 2 days, even after controlling for other risk factors of prolonged length of stay. These findings are similar to that of two prior studies identifying a decreased LOS during COVID, though one included only adults and the other included both adult and pediatric trauma patients [7, 24]. To our knowledge, this study is the first to confirm these findings solely in a pediatric trauma population. The shorter LOS could be due to providers discharging patients earlier to conserve resources during a pandemic [42, 43]. These findings could also potentially indicate that LOS during non-pandemic times is unnecessarily long, though further prospective studies collecting readmission data are needed to confirm this.

There are a number of limitations to this study. First, this was a multicenter study that utilized multiple trauma registries and data collectors, which could have resulted in misclassification and missing variables. Second, this study does not include every trauma center in the region, making the subset of pediatric patients in this study not fully representative of the entire trauma population in Southern California. Additionally, the centers included in this study were not children’s hospitals. Third, as this was a post hoc analysis with no prior power analysis, our lack of statistical significance could be due to a lack of power, although the population size was larger than the previous study which found a significant difference in penetrating trauma, but notably included animal bites. Next, we did not collect variables that would allow us to track changes in non-accidental trauma, which significantly increased in other studies, limiting us from fully exploring all the effects of COVID-19 on the pediatric trauma population [29, 33]. Finally, COVID-19-related restrictions persisted for many months following our study period, suggesting this manuscript data may not fully represent all potential changes surrounding the COVID-19 pandemic.

## Conclusion

This multicenter retrospective study in Southern California demonstrated similar penetrating trauma rates among pediatric trauma patients after SAH orders. However, the mean LOS was 1 day shorter compared to both the PRE and CONTROL cohorts. These findings may help trauma providers anticipate needs and allocate resources during future pandemics. In addition, future studies should be performed to evaluate if pediatric trauma LOS can be safely decreased during non-pandemic patient care.
